# Successful management of a rare radius schwannoma mimicking malignant bone tumors: A case report and literature review

**DOI:** 10.3389/fsurg.2023.1108942

**Published:** 2023-02-23

**Authors:** Nianzhe Sun, Umar Zeb Khan, Lei Zeng, Panfeng Wu, Qin Xiong, Lushan Peng, Hong Yu, Juyu Tang

**Affiliations:** ^1^ Department of Orthopedics, Hand & Microsurgery, National Clinical Research Center of Geriatric Disorders, Xiangya Hospital of Central South University, Changsha, China; ^2^ Department of Pathology, Xiangya Hospital of Central South University, Changsha, China; ^3^ Department of Radiology, Xiangya Hospital of Central South University, Changsha, China

**Keywords:** intraosseous schwannomas, radius, vascularized bone flap, microreconstruction surgery, bone tumor

## Abstract

**Background:**

Schwannomas are benign tumors originating from Schwann cells, frequently occurring in the spinal cord and peripheral nerves. Intraosseous schwannomas, a rare subset, account for approximately 0.2% of schwannomas. Intraosseous schwannomas commonly impinge the mandible, followed by the sacrum and the spine. By far, only three cases of radius intraosseous schwannomas have been reported in PubMed. The tumor was treated differently in all three cases, resulting in different outcomes.

**Case presentation:**

A 29-year-old male construction engineer who complained of a painless mass on the radial aspect of the right forearm was diagnosed with an intraosseous schwannoma of the radius based on radiography, three-dimensional computed tomography reconstruction, magnetic resonance imaging, pathological examination, and immunohistochemistry. A different surgical approach was employed to reconstruct the radial graft defect using bone microrepair techniques, resulting in more reliable bone healing and early functional recovery. Meanwhile, no clinical and radiographic findings suggestive of recurrence were observed at the 12-month follow-up.

**Conclusion:**

Vascularized bone flap transplantation combined with three-dimensional imaging reconstruction planning might yield better results for repairing small segmental bone defects of the radius caused by intraosseous schwannomas.

## Background

Intraosseous schwannomas (IOSs) are extremely rare and arise from peripheral nerve sheaths and spinal nerve roots (1). This benign tumor accounts for less than 1% of primary bone tumors and is more likely to invade the sacrum (2) and mandible (3) than other sites, including the long bones (4), vertebra (5), scapula (6), fibula (7), petrous apex (8), and skull bone (9, 10). In addition, the occurrence of IOSs at other bone sites has been reported less frequently in PubMed. Undoubtedly, several challenges might occur while treating such a disease. First, it is challenging for clinicians to distinguish between IOS and other benign osseous lesions (11). Complete imaging examination and confirmed pathological biopsy are required to establish a diagnosis, thereby increasing the economic burden on patients. Second, the primary disease might only be diagnosed when the tumor grows with local swelling, pain, neurological deficits, and pathological fractures (12). As a result, long-term slow-growing encapsulated tumors could unknowingly result in bone destruction and defects. A prolonged recovery period is required for the repair of bone deficits and fractures and postoperative rehabilitation care. Therefore, early detection and reliable bone reconstruction are important.

Minimally invasive surgical resection is the preferred treatment for IOS based on optimal resection outcomes and low recurrence rates (13). More importantly, repairing bone defects after surgical resection promotes early functional recovery. A systematic PubMed search revealed several previously reported potent options for bone reconstruction, including cement filling technology (4), autogenous vascularized or nonvascularized bone grafting technology (14–16), allogenous bone grafting (17), bone transport (Ilizarov technique) (18, 19), bone regeneration induced by a biological membrane (Masquelet technique) (20), mental augmentation with or without stem extension (21), titanium mesh (22), and a promising advancement of three-dimensional (3D) printed microporous implants (23). To the best of our knowledge, the interventions for bone defects vary depending on the lesion site, defect length, patient status, and medical therapeutic skills. Therefore, the options of bone repair for promoting bone healing and early mobilization are significant.

By far, only three cases of radius intraosseous schwannoma have been reported, wherein curettage of the bone lesion and cancellous bone was performed (24–26). Based on the tumor characteristics, long-term clinical and radiographical follow-up revealed no recurrence of the primary tumor with a satisfactory outcome. However, reliable blood supply, stress remodeling, and short-term recovery obscure previous surgical options. Herein, we aim to present a promising approach to harvesting matched vascularized bone flap grafts for a radial bone deficit, projected *via* 3D imaging reconstruction planning, which differs from existing technology. Through this report, we aim to propose a new way of repairing bone defects and suggest options that integrate multiple factors, such as bone location, size, and dysfunction.

## Case presentation

**Figure 1:**
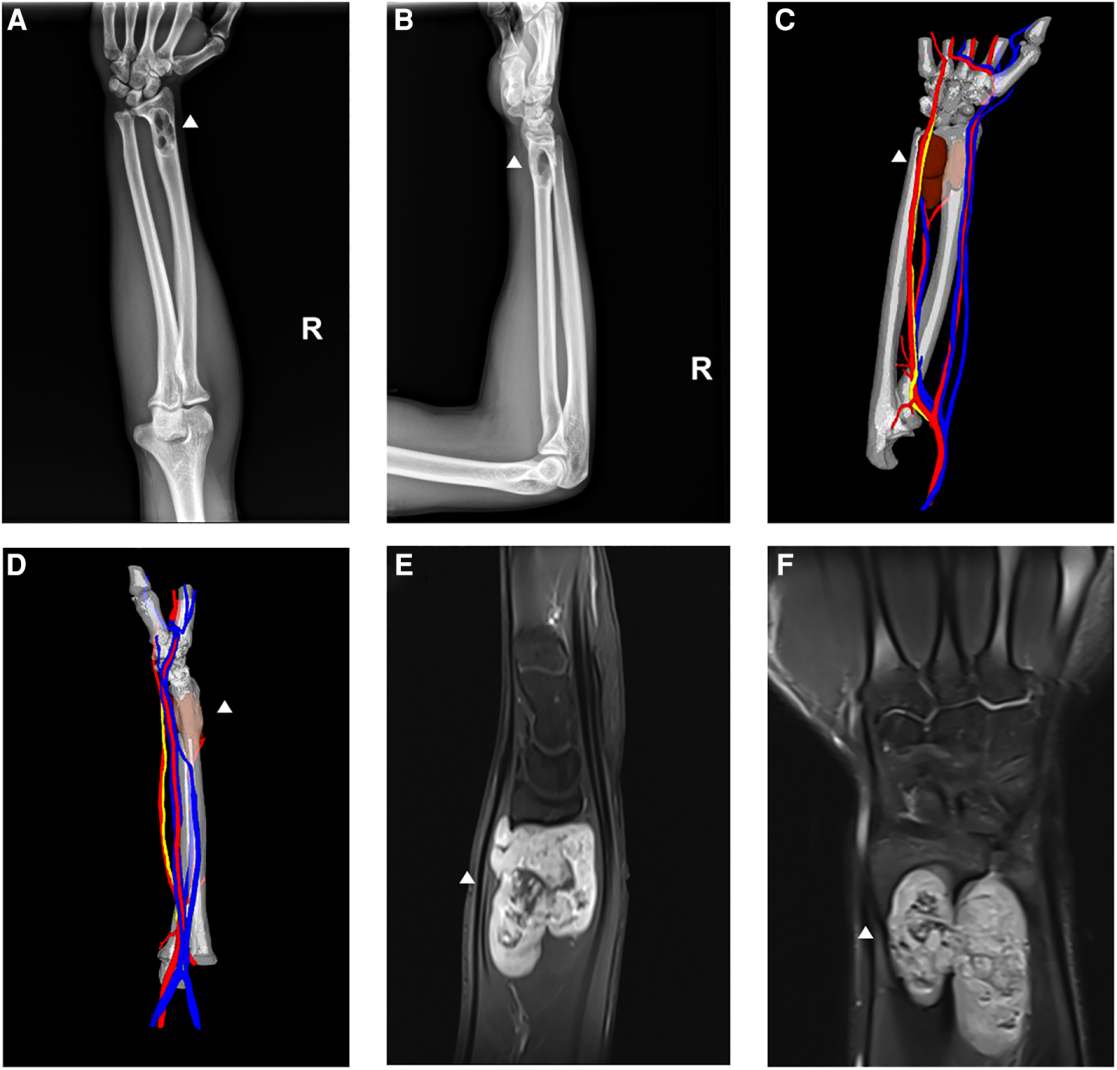
Preoperative imaging evaluation of the right forearm (white triangle indicates the location of the lesion). (**A,B**) Preoperative anteroposterior and lateral radiographs revealing the lesion in the right radius. (**C,D**) Three-dimensional computed tomography reconstruction demonstrating the positional relationship between the tumor and the bone in the anteroposterior and lateral views. (**E,F**) Lateral and anteroposterior magnetic resonance imaging indicating the invasion. The lesion measured approximately 4.8 cm × 3.7 cm × 3.6 cm, inclusive of the occupied radius.

A 29-year-old male construction engineer presented to our center with a 4-month history of a painless mass on the radial aspect of the right forearm. No significant signs and symptoms were observed. Preoperative radiography (Figure 1A,B), 3D computed tomography (CT) reconstruction (Figure 1C,D), and magnetic resonance imaging (MRI) (Figure 1E,F) were performed. The lesion could be visualized on the distal aspect of the right forearm, in the space between the ulna and the radius, invading the radius, leading to a partial radial bone defect.

**Figure 2:**
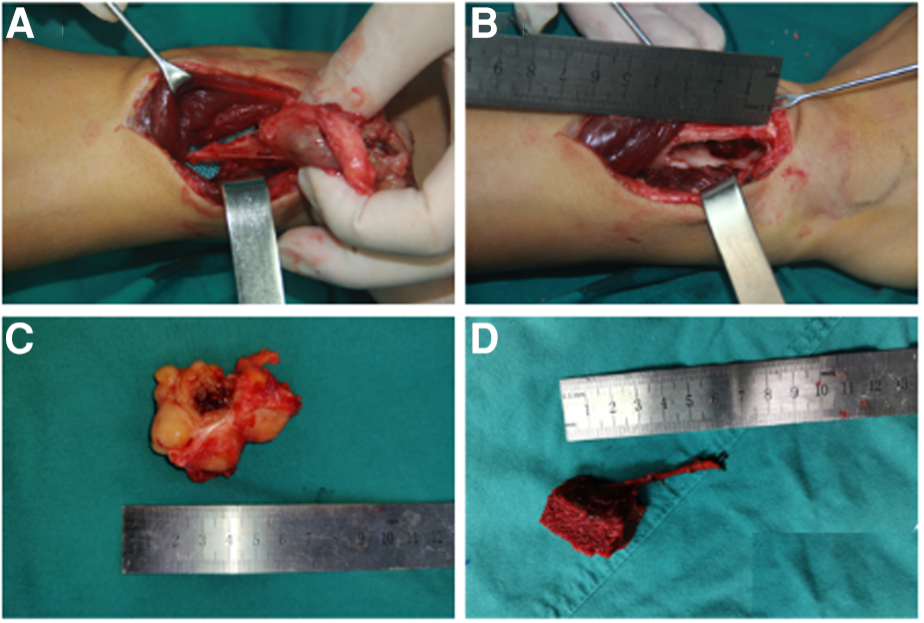
Intraoperative records of the lesion in the right forearm and free iliac bone flap. (**A**) Soft tissue mass originating from the dorsal interosseous nerve through the volar aspect of the distal radius. (**B**) A 3.5-cm-long radial bone defect through the dorsal aspect. (**C**) Macroscopic appearance of the excised lesion. (**D**) Macroscopic appearance of the vascularized free iliac bone flap for repairing the radial bone defect.

**Figure 3:**
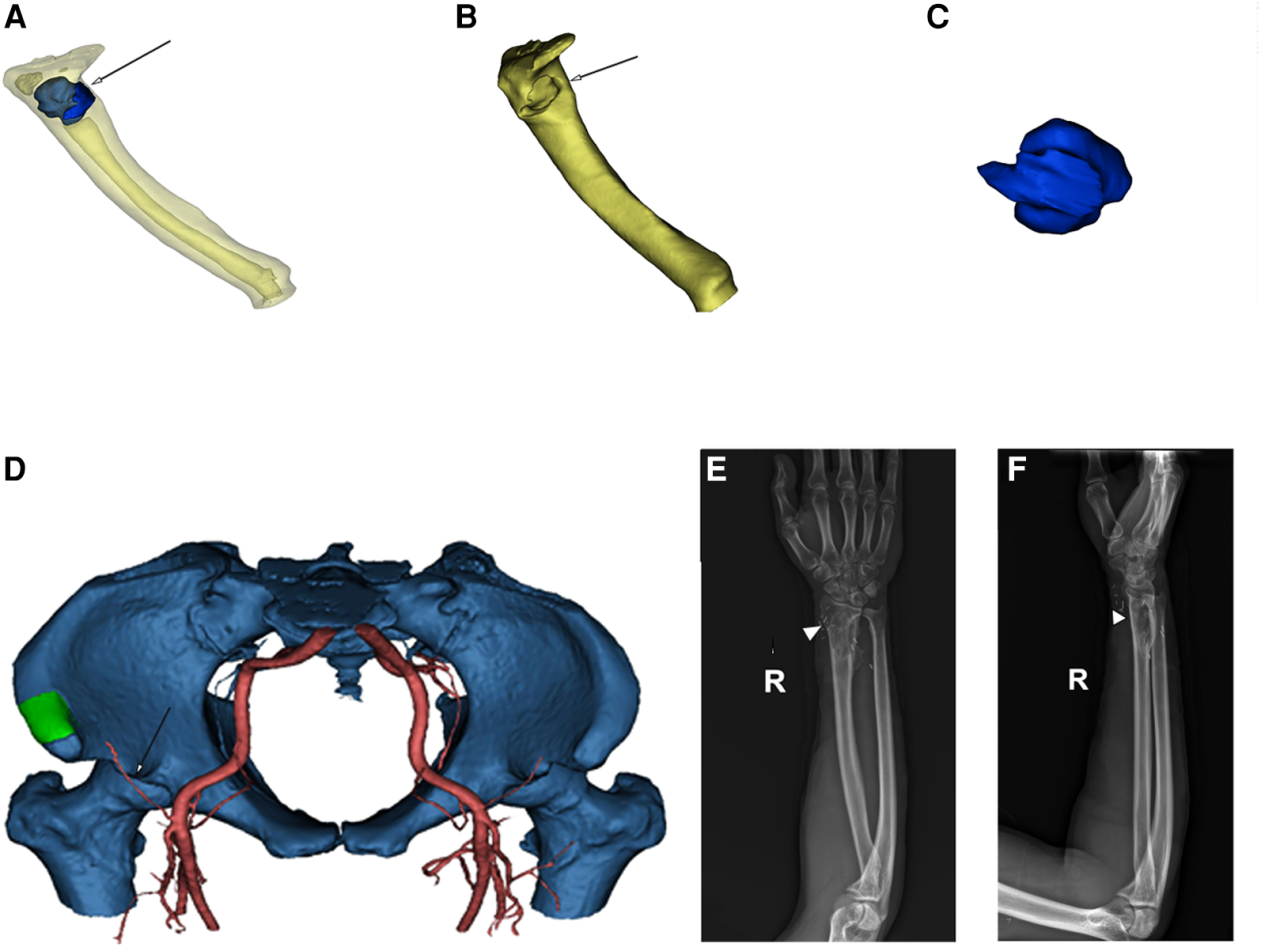
(**A**) 3D-CT reconstruction of the radius (yellow) and tumor (blue, white arrow) in the translucent view. (**B**) Radius (yellow) after tumor removal. The white arrow indicates the location of the bone defect. (**C**) Radial tumor (blue) after 3D reconstruction. (**D**) 3D reconstruction imaging (green: iliac crest, white arrow: blood supply). (**E,F**) Radiography revealing satisfactory healing (white triangle) of the radial bone defect 12 months postoperatively. 3D, three-dimensional.

**Figure 4:**
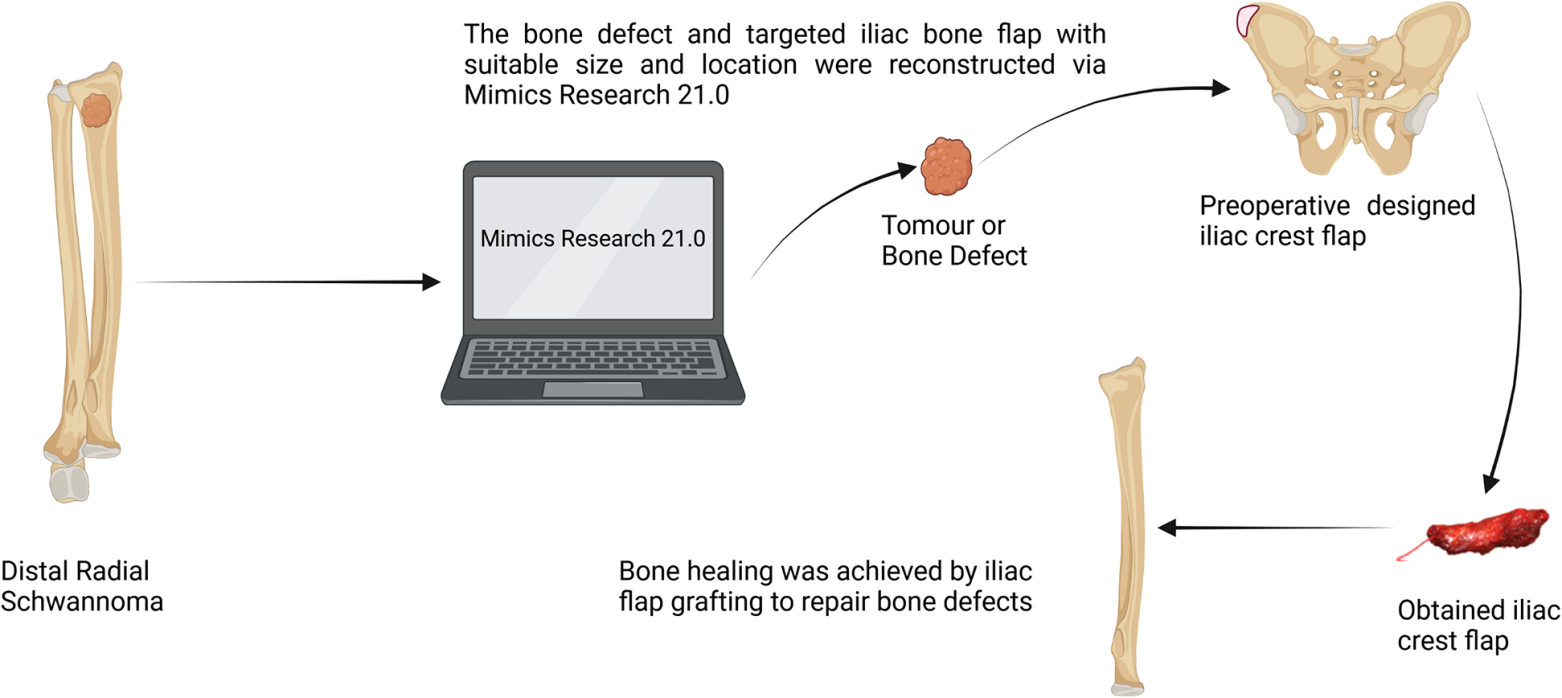
Flow chart of surgery mechanisms.

**Figure 5:**
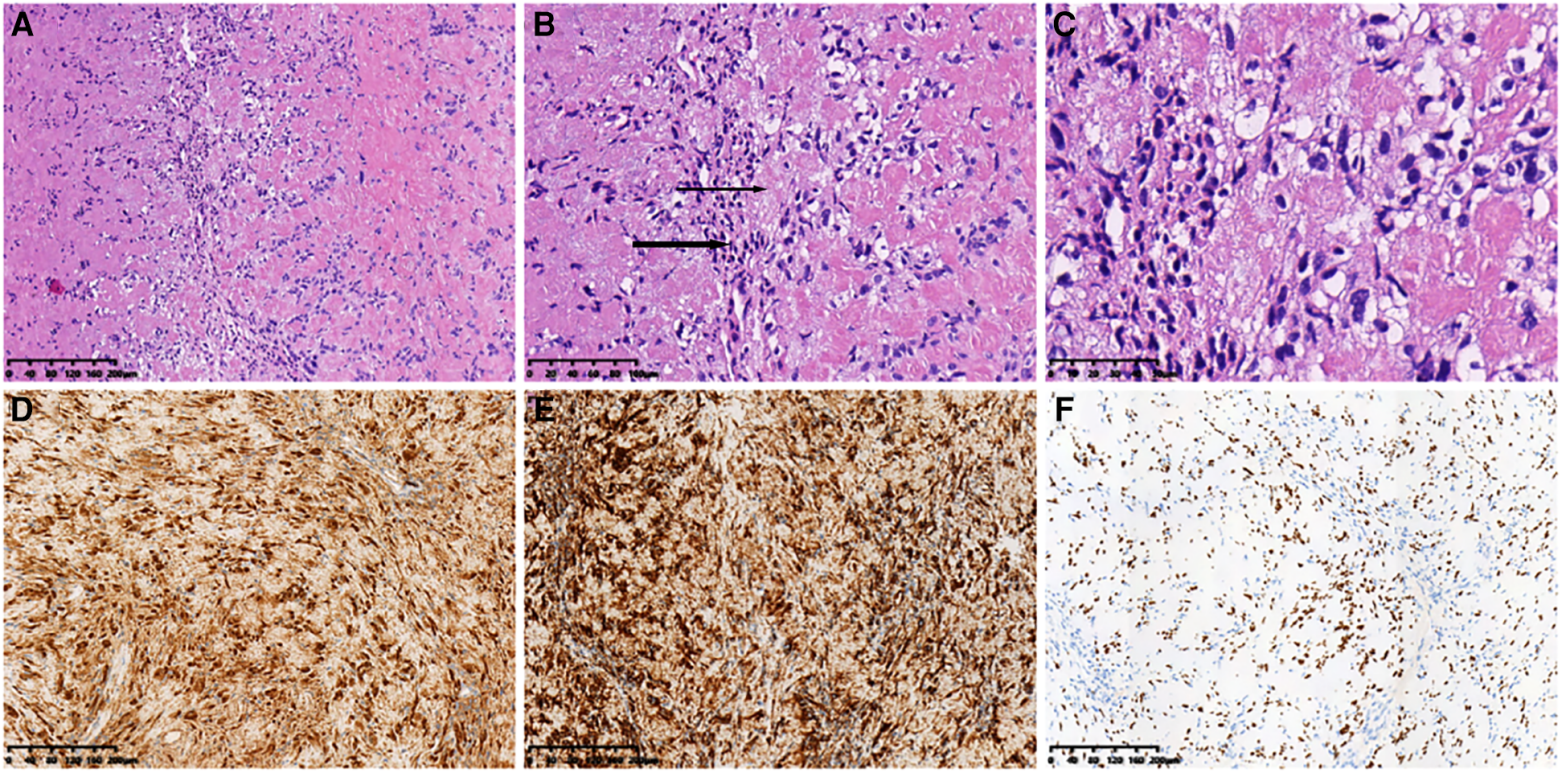
Histopathological features. (**A**) (HE ×10) Spindle tumor cells in bundles and the vortex arrangement. (**B**) (HE ×20) Alternating compact Antoni A (thick arrow) and loose Antoni B (thin Arrow) areas). (**C**) (HE ×40) Grid-like nuclear structure revealing Verocay bodies. Immunohistochemical analysis revealing positive staining for (**D**) S-100 (×10), (**E**) SOX10 (×10), and (**F**) Vimentin (×10) in most cells. HE, hematoxylin and eosin.

Surgical anatomy, pathological examination, and immunohistochemistry confirmed the diagnosis of a radial schwannoma. The operation was divided into a volar approach and a dorsal approach. In the volar approach, a straight incision was made on the right forearm from the styloid process of the radius to the tendon of the proximal biceps brachii. We identified the brachioradialis and flexor carpi radialis muscles and separated the vital structures, such as the radial nerve, artery, and vein. Finally, we separated the flexor pollicis longus and pronator muscles, exposing the palmar lesion of the distal radius. In the dorsal approach, an S-shaped incision was made on the mediodorsal wrist between the radius and the styloid process of the ulna. The space between the extensor carpi radialis brevis and the extensor digitorum was dissected, thereby exposing the musculi supinator and abductor pollicis longus, and the dorsal lesion of the distal radius was exposed. Complete tumor resection and surrounding lesion removal were performed using a combined palmar and dorsal approach. The lesion originated from the dorsal interosseous nerve *via* the volar aspect (Figure 2A). After exposing and eliminating the lesion, a 3.5-cm-long radial bone defect was identified (Figure 2B,C). Considering the patient’s need for early functional recovery and return to social activities, we used free iliac bone flaps with deep circumflex iliac vessels to repair the bone defects, and the spaces were filled with a cancellous bone to promote healing (Figure 2D). It is worth mentioning that acquiring the targeted iliac bone flap was precisely performed *via* 3D imaging reconstruction. The tumor and the radius were reconstructed using Mimics Research 21.0. Segmentally, the tumor invaded the radius (Figure 3A). The bone defect was then reconstructed (Figure 3B), and the tumor size, i.e., the size of the bone defect, was measured. The defect’s maximum length, width, and height were calculated (Figure 3C), which was the maximum length, width, and height of the iliac crest flap (Figure 3D). An abrasive drill and cancellous bone were used to ensure the iliac crest flap fit the bone defect geometrically. Also, Figure 4 was used to illustrate the surgery procedure and mechanisms briefly. The histological features comprised intermixed Antoni A and B areas combined with Verocay bodies, which were consistent with the typical clinicopathological features (Figure 5A–C) (27). The characteristic cellular area comprised spindle-shaped turnout cells arranged in a cellular, dense fascicular pattern. Immunohistochemistry for S-100 and SOX10 revealed diffuse and significant positivity, while Vimentin demonstrated weak positivity (Figure 5D–F). The Ki67 index was 1%. Other negative immunohistochemistry results were obtained for CD34, desmin, smooth muscle actin, etc. No evidence of malignancy was observed. A diagnosis of IOS was established based on these findings. Additionally, a biopsy was performed preoperatively, and its findings were consistent with the postoperative pathological diagnosis.

Follow-up radiography indicated the union of the bone gap (Figure 3E,F), and a patient-rated wrist evaluation score of 5 was recorded after 12 months. The range of motion of the right wrist significantly improved compared with the preoperative status (see the Supplementary Material). Meanwhile, no clinical and radiography findings suggestive of recurrence were observed at the 12-month follow-up.

## Discussion and conclusion

IOS is extremely rare, with an incidence of <0.2%, and it frequently occurs in the sacrum and mandible compared with other sites (2). Gordon (28) reported that most intraosseous nerves, such as the radial or fibula nerve, are nonmyelinated and only participate in vasomotor functions, which are different from the mandibular nerve originating from the trigeminal nerve. This might explain why schwannomas generally occur in the sacrum and mandible and not the long bones of the extremity. In terms of the mechanisms of bone invasion, de la Monte et al. (29) reported three approaches, which were as follows: (1) a secondary erosion of bone caused by an extraosseous tumor; (2) an enlarged nutrient canal caused by the internal tumor growing in a dumbbell-shaped configuration; and (3) a centrally arising tumor within the bone. This might explain why the tumor is more likely to occur in and invades the mandible, sacrum, and vertebrae than other bones.

Generally, the symptoms associated with IOS are mild until the tumor grows larger and are associated with pain and/or swelling. Conventional radiography generally demonstrates a low-density mass with a relatively distinct border. CT with 3D reconstruction can demonstrate the relationship between the tumor and the surrounding tissue, thereby revealing whether the spaces are filled. MRI particularly helps confirm the internal textural characteristics of the encapsulated mass to establish a preoperative diagnosis. However, this method cannot be applied to all schwannomas (30).

There are similarities between the typical histopathological features of IOS and soft tissue schwannomas. The alternating Antoni type A and B areas comprise spindle cells in a fence-like arrangement with common pleomorphic nuclei and rare mitotic figures. Meanwhile, large schwannomas measure >8 cm with hemorrhage or necrosis, resulting in cystic degeneration (31). The oncological characteristics might be more associated with its susceptibility to recurrence and the prognostic outcome. In a retrospective study based on immunohistochemical data, Li et al. (32) reported that the diagnostic and prognostic factors vary across different subtypes of spinal schwannomas. In that study, the factors, including positive-P53, Ki67 labeling index >5%, and negative-S100, were indicators for postoperative recurrence or poor prognosis. Furthermore, Karamchandani et al. (33) reported that SOX10 is more expressed than S100 in peripheral nerve sheath tumors, particularly IOS. Hence, multiple biopsies and immunohistochemical studies of the tumor combined with preoperative imaging are significant for implementing surgery and predicting the prognosis.

To the best of our knowledge, only three cases of similar lesions in the radius have been previously reported, wherein curettage of the bone lesion and cancellous bone was performed (see Table 1) (24–26). Meanwhile, surgical resection remains the gold standard and first-line treatment strategy for schwannomas, whereas chemotherapy and radiotherapy have little significance (32). However, complete resection and precise bone reconstruction with reliable vascularized bone grafts require advanced management. Herein, we present a promising approach to harvest matched vascularized bone flap grafts for a radial bone deficit, projected *via* 3D imaging reconstruction planning, which differed from existing technology. High-quality bone union and early functional recovery make this approach promising. Finally, no clinical and radiographic signs suggestive of recurrence were observed at the follow-up.

**Table 1:** Summary of all published cases of radial intraosseous schwannomas.

Number	Authors	Published year	Size (cm)	Surgical options	Follow-up time (months)	Recurrence yes/no
1	Dabrowski (24)	1976	NA	NA	NA	NA
2	Giné et al. (25)	2000	5 × 3 × 3	Curettage, cancellous iliac crest bone	30	No
3	Bağci et al. (26)	2010	10	Curettage, hemorrhagic elastic tissue with small bone particles	45	No

NA, not applicable.

Knight et al. (34) reported six benign solitary schwannomas in muscles and bones; however, in their review of 234 cases, the details on the invaded bones and treatment methods were not elaborated, which might not be the focus of this report. Meanwhile, Giné et al. (25) reported the second case of an IOS in the radius, wherein curettage was performed, and cancellous bone from the iliac crest was filled in the bone lesion, the same as that reported in the third case by Bağci et al. in 2010 (26). Long-term immobilization and bone union are no longer advised approaches. Unfortunately, the specifics of the first-documented radial IOS have been lost. Additionally, in the last report of a tumor measuring 10 cm, curettage was performed, and hemorrhagic elastic tissue with small bone particles was used to fill the bone deficit. Overall, we consider this case report to be the first to successfully use preoperative 3D imaging reconstruction to obtain a vascularized iliac bone flap to repair a radial IOS.

In conclusion, bone abnormalities are commonly based on the aforementioned potential theories and tumor characteristics. Therefore, it is critical to consider how to select various approaches to repair bone defects caused by a tumor, as in this case.

## Data Availability

The original contributions presented in the study are included in the article/**Supplementary Material**, further inquiries can be directed to the corresponding author.
