# Supplementary material for: Successful management of a rare radius schwannoma mimicking malignant bone tumors: A case report and literature review
Source: Front Surg. 2023 Feb 23;10:1108942. doi: 10.3389/fsurg.2023.1108942 (PMC9996063; doi:10.3389/fsurg.2023.1108942)
Supplement: Supplementary file 1 [file Datasheet1.docx]

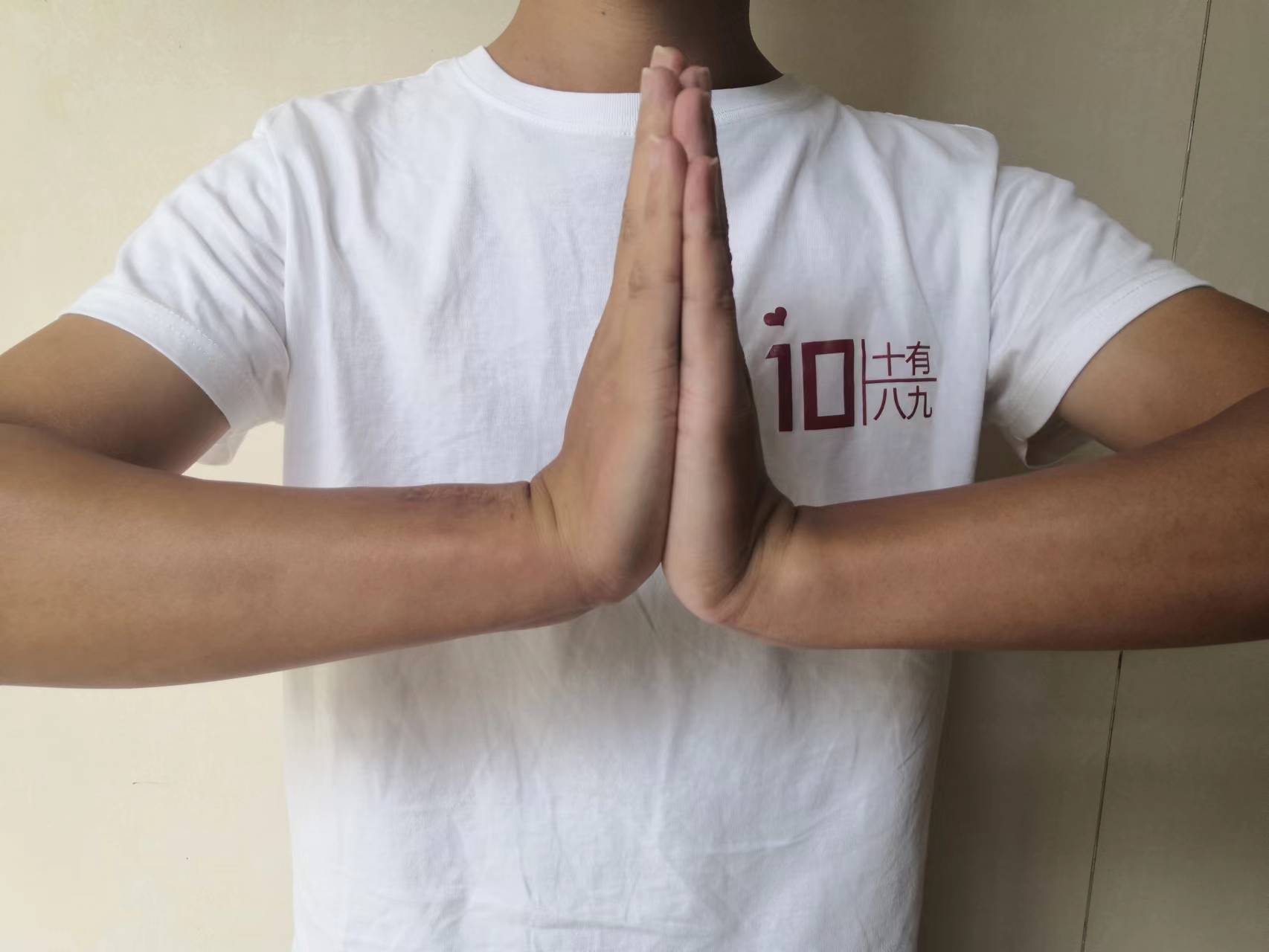

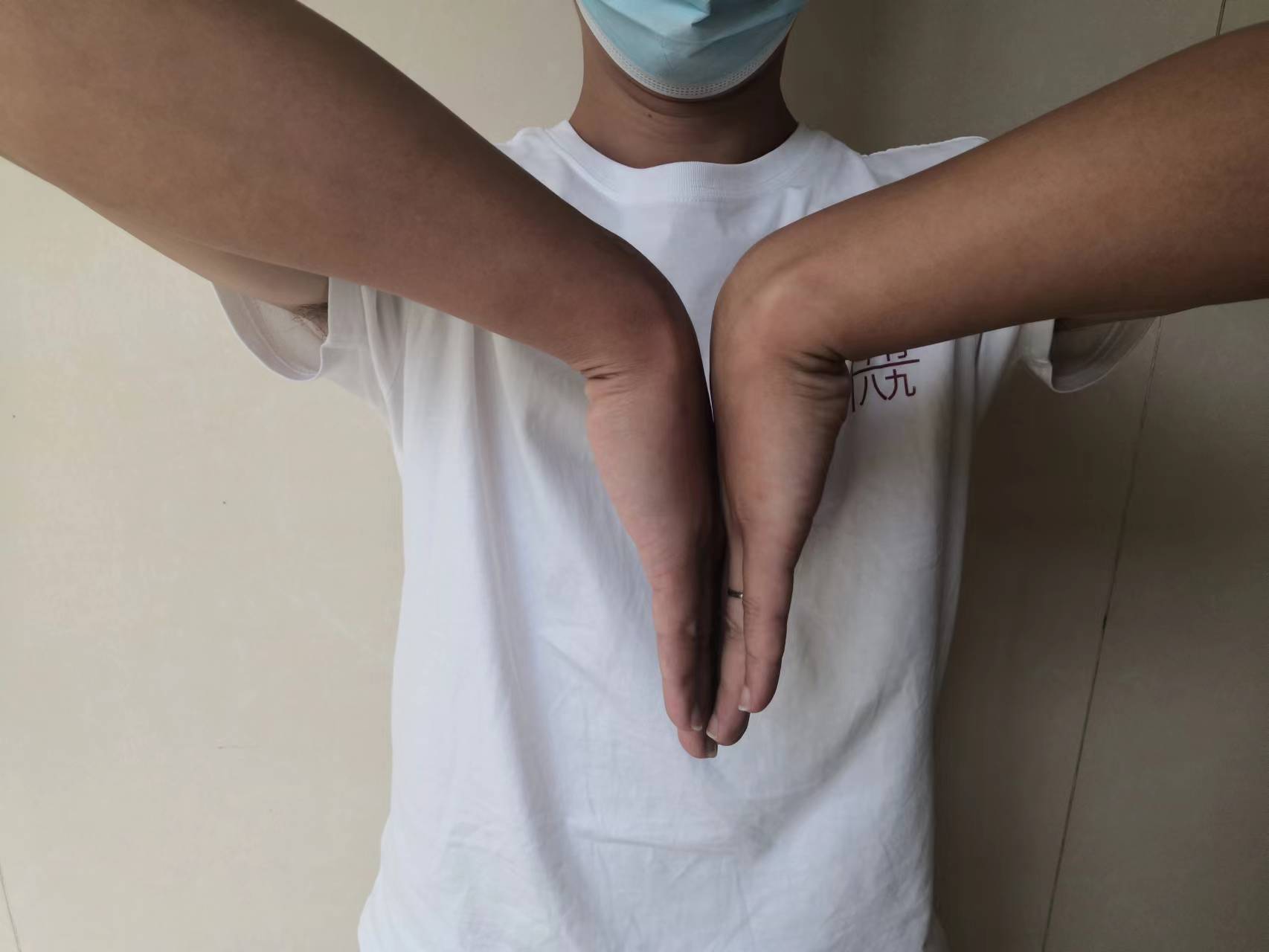


**Supplementary Material:** The patient's bilateral wrist flexion range is basically the same at 10 months postoperatively.
